# Caring Near and Far by Connecting Community-Based Clients and Family Member/Friend Caregivers Using Passive Remote Monitoring: Protocol for a Pragmatic Randomized Controlled Trial

**DOI:** 10.2196/15027

**Published:** 2020-01-10

**Authors:** Lorie Donelle, Sandra Regan, Michael Kerr, Merrick Zwarenstein, Michael Bauer, Grace Warner, Wanrudee Isaranuwatchai, Aleksandra Zecevic, Elizabeth Borycki, Dorothy Forbes, Lori Weeks, Bev Leipert, Emily Read

**Affiliations:** 1 Arthur Labatt Family School of Nursing Western University London, ON Canada; 2 Department of Family Medicine, Epidemiology and Biostatistics Western University London, ON Canada; 3 Department of Computer Science Western University London, ON Canada; 4 School of Occupational Therapy Dalhousie University Halifax, NS Canada; 5 Institute of Health Policy, Management and Evaluation University of Toronto Toronto, ON Canada; 6 School of Health Studies Western University London, ON Canada; 7 School of Health Information Science University of Victoria Victoria, BC Canada; 8 Faculty of Nursing University of Alberta Edmonton, AB Canada; 9 School of Nursing Dalhousie University Halifax, NS Canada; 10 Faculty of Nursing University of New Brunswick Fredericton, NB Canada

**Keywords:** home care, family caregiver, friend caregiver, remote sensor, older adults, caregiver burden

## Abstract

**Background:**

Significant chronic disease challenges exist among older adults. However, most older adults want to remain at home even if their health conditions challenge their ability to live independently. Yet publicly funded home care resources are scarce, private home care is expensive, and family/friend caregivers have limited capacity. Many older adults with chronic illness would require institutional care without the support from family member/friend caregivers. This role raises the risk of physical health problems, stress, burnout, and depression. Passive remote monitoring (RM), the use of sensors that do not require any action by the individual for the system to work, may increase the older adult’s ability to live independently while also providing support and peace of mind to both the client and the family member/friend caregiver.

**Objective:**

This paper presents the protocol of a study conducted in two provinces in Canada to investigate the impact of RM along with usual home care (the intervention) versus usual home care alone (control) on older adults with complex care. The primary outcome for this study is the occurrence of and time to events such as trips to emergency, short-term admission to the hospital, terminal admission to the hospital awaiting admission to long-term care, and direct admission to long-term care. The secondary outcomes for this study are (1) health care costs, (2) client functional status and quality of life in the home, (3) family/friend caregiver stress, and (4) family/friend caregiver functional health status.

**Methods:**

The design for this study is an unblinded pragmatic randomized controlled trial (PRCT) with two parallel arms in two geographic strata (Ontario and Nova Scotia). Quantitative and qualitative methodologies will be used to address the study objectives. This PRCT is conceptually informed by the principles of client-centered care and viewing the family as the client and aims at providing supported self-management.

**Results:**

This study is supported by the Canadian Institutes for Health Research. A primary completion date is anticipated in fall 2022.

**Conclusions:**

Findings from this real-world rigorous randomized trial will support Canadian decision-makers, providers, and clients and their caregivers in assessing the health, well-being, and economic benefits and the social and technological challenges of integrating RM technologies to support older adults to stay in their home, including evaluating the impact on the burden of care experienced by family/friend caregivers. With an aging population, this technology may reduce institutionalization and promote safe and independent living for the elderly as long as possible.

**Trial Registration:**

International Standard Randomised Controlled Trial Number (ISRCTN) 79884651; http://www.isrctn.com/ISRCTN79884651

**International Registered Report Identifier (IRRID):**

DERR1-10.2196/15027

## Introduction

Older adult Canadians (aged 65 years and older) experience significant chronic disease challenges [1], yet most older adults want to remain at home even if their health conditions challenge their ability to live independently [2].

There is evidence that “...when home care is appropriately managed and properly integrated into the health care system, it can improve the health and well-being of many seniors and their families and reduce the costs of care in hospitals and long-term care facilities” [3]. However, evidence suggests that there is room for improvement in current home care service delivery. Recent studies showed that 25% of Canadian older adults receive only partial home care services and experience unmet home care needs that create a cascade of events resulting in deterioration of older adults’ health and the need for institutionalized care [2,4]. Older adults want to be assured that their health care needs can and will be effectively addressed [5].

Part of the challenge in caring for older adults in their homes lies in providing suitable home care services and ensuring that older adults can safely follow their established treatment plan (eg, adherence to medication protocol). From a health care perspective, we have yet to feel the full effects of the impact of older adult health care challenges [6]. The transfer of health care services to the home setting is a strategic move intended to accommodate the desire of many older adults to remain at home and minimize acute care costs [7]. Yet home care resources are tenuous due, in part, to challenges in attracting and sustaining a work force (eg, nurses) that will be paid lower wages than in the hospital sector [3,5].

Deficiencies in availability of publicly funded home care means that family/friend caregivers are increasingly called on to play a larger role in the care of older adults within the home setting [3]. As the care needs of older adults increases, the average number of hours of care provided by family/friend caregivers increases significantly in contrast to the limited publicly funded home care service provision [3]. There is growing recognition that the majority of older adults would not be able to stay in their homes without the support from family/friend caregivers [5,8]. Family/friend caregivers in Canada are providing unpaid care to approximately 97% of home care recipients [8]. A Statistics Canada report indicated that in 2012 family/friend caregiving was provided by more than 8 million Canadians [9] at a conservative cost estimate of $25 billion (US $19 billion) in health care service [10]. These spouses, adult children, friends, and neighbors [8] provide transportation; prepare meals; clean and maintain the home; schedule and coordinate appointments; advocate for care services; manage finances; help with medical treatment; and provide emotional support and personal care such as bathing, feeding, and toileting [11]. The presence (physically or virtually) of family/friend caregivers is key to whether or not older adults with complex care needs can remain in their own home [3,12]. Yet for the family/friend caregiver, this role creates increased risk of physical health problems, stress, burnout, and depression [12].

Family/friend caregiver stress constitutes a depletion of personal resources and is reflected by family/friend caregivers’ distress, anger, or depression as a consequence of an accumulation of tasks and responsibilities that impacts their sense of independence and freedom [13]. A study by Duxbury et al [6] of employed family caregivers (eg, spouse, adult child) in Canada found that the average caregiver maintained their supportive role for about 5 years; many taking on the role because they cared deeply about their family member. However, approximately 40% of family/friend caregivers reported that they assumed the caregiver role by default; there was no one else to take on the responsibility for care. There are significant costs to Canadian family/friend caregivers of an elderly family member related to altered work patterns, loss of career advancement, and increased use of Canada’s health care system [6,8,14]. Family/friend caregivers are also at risk of financial strain associated with poorer physical and mental health, greater work-life conflict, increased workplace absenteeism, lower job satisfaction, and a higher number of visits to the emergency room and hospital. Fast et al [14] estimated an annual loss of income of $336.8 million (US $256 million) is associated with family/friend caregiver employment disruptions.

Health information technology and specifically remote monitoring (RM) technologies can support older adults to remain in their homes [15]. Remote technologies act by notifying, in this case, a family member or friend of a possible untoward incident (ie, a fall, failure to take medications, wandering). Active RM monitoring applications require individual participation, such as pushing a button, whereas passive RM technologies such as sensors do not require any action by the individual for the system to work. Active and passive RM can increase client confidence in their self-health care and in their ability to live independently at home [16]. RM is especially helpful in tracking behaviors of older adults with cognitive decline (eg, forgetting to take medications) and allowing caregivers to intervene quickly [15]. These technologies can also benefit home-based older adults and their family/friend caregivers in the short-term by increasing communication and collaboration between and among all stakeholders [15].

We are proposing to develop and evaluate a technology-enabled RM model of home care (RMHC) to address current gaps in home care for older adults requiring the kind of complex care that includes (1) stretched health human resources, (2) partial home care services and unmet home care needs, (3) reliance on unpaid family/friend caregivers to sustain home care services with limited support to conduct this care work, (4) a chronic disease model of health care that assumes and expects client and caregiver ability for self-care, (5) lack of direct support for or involvement of family caregivers in new models of home care, and a (6) lack of innovative strategies to expand home care. RMHC is enabled by passive RM technologies (eg, sensors and cameras) and is conceptually informed by the principles of client-centered care, family as client, supported self-management, and stakeholder collaboration [4,5,17,18].

The purpose of this study is to examine the use of passive RM technologies in the home as a means of supporting older adults to safely remain in their home and avoid or delay the need for higher levels of care.

## Methods

### Study Design

This 4-year study is an unblinded pragmatic randomized controlled trial (PRCT) with two parallel arms [19-22]. PRCTs are well-suited to supporting decisions about complex interventions tested in the real world when comparing with usual care [23,24]. The objective of the study is to examine the effectiveness of the RMHC versus usual home care in maintaining the client in their place of residence and delaying or preventing admission to higher levels of care such as hospitalization or long-term care.

This study builds on the findings of previous pilot studies conducted in the provinces of New Brunswick, Alberta, Nova Scotia, and British Columbia. The primary aims of this study are to test whether (1) RM along with usual home care (the intervention) versus usual home care alone (control) allow older adults with complex care needs to remain in their home longer and delay or avoid admission to higher levels of care (eg, hospitalization and long-term care), (2) intervention is cost effective, and (3) intervention will improve the quality of life for clients and family/friend caregivers. The primary outcome for this study is the occurrence of and time to events such as terminal admission to hospital awaiting admission to long-term care and direct admission to long-term care. The secondary outcomes for this study are (1) health care costs, (2) client functional status and quality of life in the home, (3) family/friend caregiver stress, and (4) family/friend caregiver functional health status.

### Setting

The PRCT will take place in two study sites: the provinces of Nova Scotia and Ontario.

### Selection Criteria

PRCTs are meant to capture real-world situations and clients, therefore we have purposely selected broad inclusion criteria for this study (Textbox 1). To be assessed as at risk for higher levels of care, the clinical provider identifies that within the next 12 months the client is likely to be admitted to long-term care. For this research, participants must be recruited in client-caregiver dyads because the RM technology requires that older adults have a family member/friend caregiver who is willing to receive system notifications. These criteria were developed in collaboration with the regional health care partners.

**Participant recruitment selection criteria.**

Inclusion criteria:

- Aged 65 years and older
- Assessed as requiring complex publicly funded home care
- At risk for admission to a higher level of care
- Have a caregiver (family member or friend or neighbor) willing to receive notifications from the remote monitoring system
- Willing to have remote monitoring technology installed in their home if they are randomized to the intervention group
- Able to read/write English or French
- Have decisional capacity to consent or have a substitute decision-maker consent to participate in study

Exclusion criteria:

- Assessed for immediate admission for higher level of care
- Lack a family/friend caregiver
- Not competent to consent to participate in study or do not have a substitute decision-maker for consent

### Sample Size Calculation

The sample size calculation is based on data from one health region included in the study to estimate the study’s main outcome (time to higher levels of care). The following criteria were used to estimate sample size: the total institutionalization proportion among controls being equal to 0.41 and the proportion or the experimental subjects at 0.27 (ie, a 34% reduction compared to controls); a 10% dropout and/or lost to follow-up rate; a power of 80% and a statistical significance level of alpha=.05. This resulted in an estimate of 160 participants for the intervention group and 320 for the control group for a total target study size of 480 participants across the study sites [25,26]. We opted to increase the study power by using a 2:1 ratio of controls to intervention subjects due to the cost of the intervention.

### Recruitment

Participant enrollment in the PRCT is supported by the respective provincial regional authority. Home care recipient participants will be assessed by the case coordinator or case manager in the regional authority against the inclusion and exclusion criteria (see Textbox 1).

Consent to participate in the study will be obtained at the first meeting with the researcher and the patient and family/friend caregiver. Once consent is obtained from the participants (client and their family/friend caregiver), research staff will collect baseline data before initiating random allocation into control and intervention groups. Using block randomization, participants will receive either usual home care or RM with usual home care [27]. Randomization will occur as follows:

- Twelve ping pong balls will be placed in a container
- Four of each of the ping pong balls will be numbered 1 to 3
- Each block of 3 participants will be numbered sequentially from 1 to 3 as they appear on the master recruitment list
- A ping pong ball will be pulled from a container and whatever number that ball is will correspond to the numbered participant; it is that participant who will receive the intervention
- The remaining 2 participants will be in the control arm (no intervention)

Allocation bias will be addressed through allocation concealment; neither the case coordinator/case manager in the home care agency or research staff will know to which group the individual will be assigned a priori.

### Control and Intervention Groups

#### Control

Clients will receive usual publicly funded home care services provided by their provincial or regional home care agency including but not limited to home visits by assistive personnel for activities of daily living, nursing care, and other supports deemed necessary by the home care case coordinator or case manager. In both study sites, home care assessments are conducted by the regional authority case coordinator or case manager. Once the assessment is completed and care services are decided, service delivery is provided by a contracted home care agency.

#### Intervention

Clients will receive RMHC in addition to usual home care services. The intervention group will receive passive RM sensors to detect a combination of the following behaviors/movement patterns: medication administration, opening refrigerator/cupboards, getting in and out of bed, movement in the bathroom, use of exit doors, movement detection, and for observation (cameras). The study technology partner, CareLink Advantage (www.carelinkadvantage.ca), will cover the equipment and monitoring costs. Once enrolled in the study, clients and their family/friend caregivers will have a home visit by the technology partner (or designate). They will receive written and verbal overview of the various RM options. Based on the assessment by the technology partner along with client and family/friend caregiver preferences, the RM options will be customized and implemented. The intervention will be offered to the client for 12 months at which time the client will be transitioned to usual care. Notifications of atypical events (eg, missed medication, atypical length of time in bed) will be sent to the family/friend caregiver via either email, text message, or phone call. Action, based on the notification, may include a telephone call to prompt the client or check on the client’s safety, deployment of assistive home care supports, or emergency action (eg, ambulance). During the study, researchers will not receive any notifications. However, at the end of the study notification patterns and trends will be analyzed.

### Data Collection

Prior to initiating the PRCT, all research team staff in Nova Scotia and Ontario and study site care coordinators/case managers will receive training on the study protocol and data collection measures. In addition, research staff and home care agency staff will be educated about the RM equipment by the industry partner (CareLink Advantage).

#### Phase I: Survey Data Collection

Researchers will meet with clients and family/friend caregivers in the home of the client or by phone at baseline, 6 months, and 12 months to collect data. It is expected the interviews will last no more than one hour. Paper format questionnaires including standardized instruments and researcher-developed questions will be used to collect data at each time point (see Measures). Data on reasons for leaving the study will also be collected. A demographic form will be used to collect basic demographics including age, education, sex, marital status, income, and individuals’ provincial health insurance number. The provincial health insurance number will be used for data linkage to provincial administrative health care databases in order to conduct the economic evaluation.

#### Phase II: Administrative Data Linkage

To address the primary outcome of the study, data will be obtained from the regional health authorities related to the occurrence of and time to events such as trips to emergency, short-term admission to hospital, terminal admission to hospital, and direct admission to long-term care.

#### Phase III: Qualitative Interviews and Focus Groups

Qualitative data will be collected in the form of semistructured interviews to understand the perspectives of clients, family/friend caregivers, health care professionals, and health care decision-makers on the use of RM in the home. Eight to 12 individuals each of clients, family/friend caregivers, health care professionals, and health care decision-makers will be interviewed. Interviews will be audio recorded and transcribed verbatim. To ensure accuracy of the transcribed data, a random selection of transcripts will be validated with audio recording. Interviews will focus on understanding views on the technology itself including acceptability/resistance (eg, privacy), the model of care (eg, client-centered), enhancements/improvements, and benefits of the model for clients, family/friend caregivers, health care professionals, and health care decision-makers. These data will assist with understanding implementation and evaluation of the model, inform modifications to the model for potential scale-up, and integrate end user perspectives into model refinements.

### Measures

The primary outcome of the study is the occurrence of and time to leaving one’s home for a higher level of care such as long-term care. This will be assessed using the following variables available in the Discharge Abstract Database for Ontario and Nova Scotia: terminal admission to hospital (yes/no) and direct admission to long-term care (yes/no).

Secondary outcomes are described below along with their specific measures. All standardized instruments have been validated in previous studies. Different versions of researcher-developed instruments were developed for clients and family/friend caregivers.

#### Health Care Costs

Health care cost to the Ministries of Health (ie, hospitalization, emergency room visits, and home care services) will be captured from administrative databases (from provincial and regional authority databases).

#### Older Adult Functional Status and Quality of Life

Older adults’ functional status, mood, well-being, and social supports will be assessed using the following:

- Hospital Admission Risk Profile [28]
- Participant self-reported perception of safety and quality of care (researcher-developed)
- Mini-Mental State Examination [29]
- Satisfaction with RM (researcher-developed)
- Older People’s Quality of Life Questionnaire [30]

#### Family and Friend Caregiver Functional Status and Quality of Life

Measures include adapted versions of the following:

- Selected Caregiver Assessment Measures [31]
- California Caregiver Resource Centers Uniform Assessment Tool [32]
- Zarit Burden Interview [33]
- Positive Aspects of Caring [34]
- Stanford Presenteeism Scale [35]
- Todtman Financial Impact Scale [36]
- Satisfaction with in-home monitoring assessment (researcher-developed)

#### Remote Monitoring Data

Sensor notifications are atypical events (eg, missed medication, lack of movement from the bed) of client’s activities of daily living that will trigger a message sent to a family/friend caregiver. Currently, all system (ie, client) activity is logged and viewable on a dashboard available to authorized individuals via computer or on a mobile device. As well, a history of the clients’ activity/nonactivity is logged (eg, fridge door opened, bathroom motion idle). The system has a search function such that client activity can be determined by sensor (eg, bed, medication) over several months to determine emerging and changing patterns. Patterns will be identified by the application of algorithms. Alternatively, all activity of a day can be viewed in chronological order to generate a description of the clients’ daily behavior patterns. The notification data has the potential to form a baseline of participant (client) behavior in the home, and trends or models of a participant’s usual activity or behavior can be established and then used to identify changes. Data collected from camera sensors will not be assessed.

### Data Analysis

#### Quantitative Data

Quantitative data will be analyzed at baseline, with subsequent analysis of primary/secondary outcomes at 6 months and 12 months. Using SPSS Statistics version 25 (IBM Corp) statistical software, descriptive statistics will provide a profile of study participants including demographics, current diagnoses, length of home care before the study, and model of care (RMHC versus usual care). The provisional effect of the intervention on the primary outcome will be tested using a prospective chi-square test of independence (continuity-corrected) between the experimental and control groups (Fisher exact test). Repeated measures analysis of variance will measure mean differences between groups on continuous variables. We intend to use multivariate survival analyses methods using a relevant time dependent outcome based on the ability to accurately track the main event of interest (ie, institutionalization) [24]. We will conduct within and between province analyses on secondary outcomes. Subgroup analyses will include examining differences based on sex (male/female) in both clients and family/friend caregivers.

#### Notification Data Analysis

Notification data from RMHC sensors will be analyzed for patterns and trends of clients’ activity in the home. Data will be downloaded from the technology provider database quarterly and analyzed by the researchers. Exploratory pattern analysis will include reviewing each individual’s pattern of notifications. In addition, data will be aggregated to examine patterns that might inform understanding of the population (eg, common issues such as medication delay, sleep/wake patterns). These data will provide broader insights about daily patterns of older adults living in their home that may lead to other interventions to support home care service planning.

#### Economic Analysis

An economic analysis will be undertaken by the study health economists to examine health care costs from the perspective of the health system. The objective of the economic analysis is to compare the costs associated the RMHC model against those of the usual publicly funded home care received by control group participants. The economic analysis will be conducted using primary data from the study and secondary data from the administrative databases (from provincial and regional health authority databases—ie, the Ontario Institute for Clinical Evaluative Sciences, Health Data Nova Scotia ) for costs associated with older adult health service use (ie, hospitalization, emergency room visits, and home care services) along with costs associated with home care delivery. The costs of the delivery (service fees for installation and monthly maintenance) of the RM intervention will also be included. With data on health service use, costs for each older adult participant will be calculated by multiplying the individual resource use (from the study and home care database) with unit costs (from provincial standard costing sources such as the Schedule of Benefits [36-39]). We will analyze the total cost variable as a dependent variable, using regression, to estimate the difference in expected health care cost between the RMHC model of care and usual care. The intervention variable will be the primary independent variable, and the model will adjust for previously mentioned potential confounding variables. Ordinary least squares model produces unbiased estimates even if the data are skewed [40]. Subgroup analyses (eg, by sites, by sex) will be explored.

#### Qualitative Analysis

Qualitative data analysis will be conducted using an interpretive description approach. Semistructured interviews will be used. The interview guide will be developed based on data discovered from the baseline and 6- and 12-month interviews. Clients and caregivers will be interviewed separately. Interpretive description is an inductive analytic approach designed to analyze clinical phenomena with the intent of identifying thematic patterns within participants’ experiences to inform clinical understanding and application [41]. The focus of this approach is not only intent on describing phenomena but on developing explanations which have meaningful application thus aligning this approach to data analysis with identifying and addressing practice issues, informing the development of policy and planning of home care services. Using NVivo version 10 (QSR International Pty Ltd) qualitative software, at least two researchers will independently code the data to ensure reliability in the coding. Data will be analyzed for emergent themes relevant to the study outcomes and to inform scale-up of the RMHC model.

## Results

The end of data collection for the primary outcome (occurrence of and time to terminal admission to hospital awaiting admission to long-term care and direct admission to long-term care) will be winter 2020. Data collection for the secondary outcome (health service use) will continue for up to 5 years after the 12-month participant interview. Qualitative data will be collected during study years 3 through 5.

## Discussion

### Impact

Research evidence indicates that the real challenge in using RM technologies is not about the number and placement of sensors in the home but rather the ability to make sense of and respond in a timely manner to the data streams from clients [42]. To be effective, the notification data could be tailored to and uniquely presented to providers, older adults, and family/friend caregivers. RMHC notification to family/friend caregivers with the option to share this information with home care service providers (1) enhance family/friend caregiver support, (2) inform decision-making regarding client care to determine safety (eg, proper medication administration, falls), and (3) generate observations of daily living from client data for historical analysis for trending, modeling, and prediction [43]. With historical data, there is potential to model older adults’ behavior to identify patterns that can be used to identify changes in behavior over time.

In the RMHC model, we redefine the term client to include both older adults in the home and their family/friend caregivers [5,12]. Although family/friend caregivers play an integral role as part of the care team to support older adults in the home, health care assessments remain largely focused on the needs of the individual client. The extended health care responsibilities taken up by older adults and their family/friend caregivers create care work. Family/friend caregivers in the RMHC model will also be recognized as clients with unique care needs [5,12]. Many family/friend caregivers also experience a decline in their health but continue to provide care. In light of this, researchers have concluded that a reasonable outcome for family/friend caregivers is one where caregiver stress is held constant [44]. Minimizing family/friend caregiver stress in light of the evolving care challenges of older adults in the home may be unrealistic [44]. In the RMHC model careful attention is given to the caregiver experience.

### Conclusion

The societal impact of RMHC intervention may contribute to resolving the challenges of limited health human resources within home care and create greater benefits for family/friend caregivers. Collecting data on older adults’ behaviors creates the opportunity to efficiently monitor and address the needs of home care clients but also to aggregate data into large datasets to be analyzed and used to inform the development of best practices for older adults with complex care needs and their family/friend caregivers within the home care setting.

## Abbreviations

PRCT: pragmatic randomized controlled trial
RM: remote monitoring
RMHC: remote monitoring model of home care
